# Digging into the Cause of Abnormal Patellar Kinematics After Open-Wedge High Tibial Osteotomy via a Quantitative Study on In Vivo Soft Tissue Functional Changes

**DOI:** 10.3390/bioengineering12020123

**Published:** 2025-01-28

**Authors:** Zheng Jiang, Nan Zheng, Axiang He, Guoqiang Zhang, Weiming Lin, Yang Qu, Tsung-Yuan Tsai, Wanjun Liu, Yanjie Mao

**Affiliations:** 1 Department of Orthopedics, Shanghai Sixth People’s Hospital Affiliated to Shanghai Jiao Tong University School of Medicine, Shanghai 200235, China; 122725912252@sjtu.edu.cn (Z.J.); zsshu123@sina.com (A.H.); leon1126@sjtu.edu.cn (W.L.); ytwanjun@163.com (W.L.); 2 School of Biomedical Engineering, Med-X Research Institute, Shanghai Jiao Tong University, Shanghai 200240, China; zheng_nan@sjtu.edu.cn; 3 The Fourth Medical Center, Chinese PLA General Hospital, Beijing 100853, China; gqzhang301@163.com; 4 Department of Radiology, Shanghai Sixth People’s Hospital Affiliated to Shanghai Jiao Tong University School of Medicine, Shanghai 200235, China; qyquyang@126.com

**Keywords:** osteotomy, patellar tendon, medial patellotibial ligament, medial patellofemoral ligament, force arm, patellofemoral joint, dual fluoroscopic imaging system

## Abstract

The biomechanical mechanism of postoperative patellofemoral joint (PFJ) complications after open-wedge high tibial osteotomy (OWHTO) has not been investigated. This study was to determine the length changes in the patellar tendon (PT), medial patellotibial ligament (MPTL), medial patellofemoral ligament (MPFL), and quadriceps moment arm (QMA) during staircase motion before and after OWHTO. Computed tomography (CT) scans of 15 patients’ lower extremities were used to reconstruct three-dimensional models, and magnetic resonance imaging (MRI) of the knee and hip joints was used to mark the soft tissue footprints. Then, such soft tissue lengths were quantified by a dual fluoroscopic imaging system (DFIS). Additionally, function scores were used to assess patient outcome changes. The results showed that there was a contraction of the PT after OWHTO due to its adhesion to the osteotomy site, causing PT length to be negatively correlated to the open-wedge angle. In addition, the shortening of the MPTL and QMA caused patellar instability and an imbalance in the strength of the lower extremities. Additionally, most knee function scores improved after OWHTO, except the Feller scores. Multiple methods should be considered to optimize surgical procedures, postoperative rehabilitation, and physical therapy.

## 1. Introduction

The number of knee osteoarthritis (KOA) patients increases annually in both elderly and young individuals [1]. High tibial osteotomy (HTO) is one of the mainstay treatments for early osteoarthritis and cartilage damage in the varus knee, especially in physiologically active patients presenting with medial compartment KOA [2]. Among HTO procedures, OWHTO has become the most utilized due to decreased operation difficulty and intraoperative complication rates [3], with an 87.7% long-term survival rate [4]. However, previous follow-up studies revealed that the OWHTO procedure altered the anatomy of the patellofemoral joint (PFJ) and patella kinematics, leading to 11.4–32% anterior knee pain (AKP) or clinical evidence of PFJ malalignment [5] and even degeneration of the PFJ cartilage [6,7]. These changes have adverse effects on patients’ quality of life, together with the difficulty encountered in total knee arthroplasty (TKA) after HTO [8]. Previous studies believed these adverse clinical outcomes were related to abnormal kinematics of the patella, discovering that OWHTO increased patellar translation distally and laterally, and increased patellar valgus rotation and medial tilt [9]. Therefore, there is an urgent need to clarify the biomechanical mechanism of such patellar abnormal movements after OWHTO, aiming to find treatments for PFJ complications and improve postoperative rehabilitation.

The functions of the medial patellofemoral ligament (MPFL) and medial patellotibial ligament (MPTL) have been widely investigated due to their important role in patella instability. A previous study reported that the MPFL was the primary restraint for lateral translation of the patella [10,11], whereas the MPTL and medial patellomeniscal ligament (MPML) combined to provide approximately 46% of the restraint forces against lateral patellar subluxation when the knee was at 90° of flexion. Additionally, the MPTL and MPML were responsible for 72% of patellar rotation and 92% of patellar adduction [12,13]. In addition, another cadaver study [14] indicated that the force needed to displace the patella by 1 cm was significantly less at all flexion angles when the MPTL and MPFL were both transected than when the MPTL and MPFL were intact or when either of them was reconstructed. Namely, MPFL deficiency can cause patellar instability, which further increases with a deficient MPTL. However, functional changes in the MPFL and MPTL after OWHTO have not been reported, the observation of which is essential for clarifying the mechanism of AKP or clinical evidence of PFJ malalignment.

The patellar tendon (PT), which connects the patella and tibial tubercle, is a crucial tissue related to patellar kinematics [15,16]. It was previously reported that PT length shortening was more prevalent in total knee arthroplasties (TKAs), while lengthening was more prevalent in lateral unicompartmental knee arthroplasties (UKAs), and no significant change was found in medial UKAs [16]. Gokay et al. [15] discovered that close-wedge high tibial osteotomy (CWHTO) might lead to adherence of the tendon on the proximal side of the osteotomy site. They reported that the severity of vascularization, inflammation, and fibrotic changes was evident, which resulted in shortening of the PT and patella lowering. Although it was reported that OWHTO patients were more susceptible to shortening of the PT and patella descent than CWHTO patients [17], quantitative studies on the PT functional and radiographic changes after OWHTO are still lacking.

The quadriceps moment arm (QMA) could serve as a valid way to measure the moment quadriceps forces and patellofemoral compressive forces [18] during knee extension. The quadriceps muscle generates most of the force acting on the anterior knee. Osteotomy to change limb alignment is powerful to alter the direction of the quadriceps vector [19]. In addition, the patella functions like a lever and belongs to the extensor mechanism of the knee, where the patella could be considered a sesamoid bone, improving the efficiency by increasing the QMA to the center of rotation of the knee [20]. The patella provides a fulcrum for the quadriceps muscles, where they attach a knee extension moment. Abnormalities in the structure or position of the patella can significantly reduce the ability of the quadriceps to output torque, thereby affecting normal knee flexion and extension. Therefore, increasing the QMA could reduce quadriceps tension and patellofemoral compressive forces. Clarifying QMA changes during postoperative daily activities is highly important for evaluating patients’ low extremities’ force balance.

In addition, the open-wedge angle is a surgical parameter of OWHTO that seems to be directly related to PT changes. In a retrospective review of OWHTO, patellar descent was found to be correlated with the size of the anterior bone graft, which is highly dependent on the open-wedge angle and intraoperative bone loss [21]. Both an insufficient and excessively open-wedge angle would lead to surgical failure, so it is necessary to maintain the angle to a reasonable extent.

The DFIS is currently the most accurate technology for tracking in vivo motion and has been widely used in the observation of in vivo knee joint kinematics [22,23] and the function of related ligaments during gait [24]. In addition, Feller scores are specific for evaluating the function of the patella [25], and some knee function scores like Visual Analogue Scale-Satisfaction (VAS), Western Ontario and McMaster Universities Osteoarthritis Index (WOMAC) [26], Hospital for Special Surgery (HSS), Oxford Knee Score (OKS) [27], and Short form-36 (SF-36) are widely used in follow-up studies of knee surgeries [28], serving as a supplement to our observations. Furthermore, climbing stairs is one of the most demanding activities closely related to the PFJ under weight-bearing conditions [29] and is one of the most painful and challenging activities of daily living for subjects with anterior knee pain [30]. Therefore, investigating the postoperative functional changes in soft tissues related to patellar kinematics during daily stair-climbing motion is highly clinically important.

The purposes of this study were to (1) quantify the postoperative functional changes in the PT, MPTL, MPFL, and QMA during stair climbing and compare them with those on the native side and (2) investigate the relationship between the length of the ligaments and the opening wedge angle of OWHTO, and (3) search for the treatments to deal with such adverse changes and provide inspirations for further clinical studies.

## 2. Materials and Methods

Fifteen patients (5 male and 10 female) who complied with the requirements of the Institutional Review Board (IRB No. 2024-KY-150(K)) were enrolled in the current study. A post hoc statistical benefit–volume analysis was performed using G*Power 3.1 (Universität Kiel, Kiel, Germany), and 15 OWHTO patients included in this study had effect sizes above 0.92. The patients’ average age was 56.7 ± 5.2 years, and the average body mass index was 28.4 ± 3.7 kg/m^2^. The inclusion criteria were no previous trauma or surgical history on either knee, no neuropsychiatric diseases, and age less than 65 years. All patients underwent unilateral OWHTO (seven left knees and eight right knees) due to symptomatic varus knee, which was diagnosed as early KOA (Kellgren–Lawrence grade [31] I~II), with their contralateral extremities being asymptomatic and intact. All patients followed the same postoperative rehabilitation protocol from our team.

**Figure 1:**
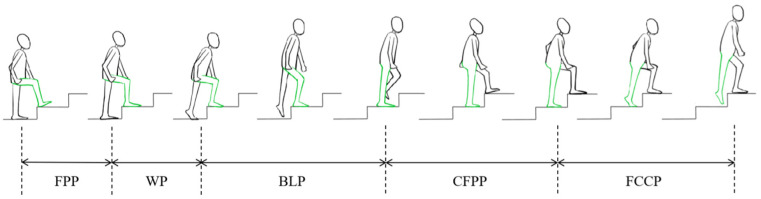
A complete gait cycle scheme during stair climbing, consisting of a support phase, including a Weight-bearing Phase (WP), a Body Lifting Phase (BLP), and a Continuous Forward Propulsion Phase (CFPP); and a swing phase, including a Foot Contour Clearance Phase (FCCP) and a Foot Placement Phase (FPP) [37]. The FPP could not be measured in the motion cycle of this study for the contiguity of stair-climbing motion.

All the equipment was provided by the National Center for Orthopedics (Shanghai, China). Each patient underwent CT scans (Siemens 64, Siemens, Berlin, Germany) of both lower limbs before and 6 months after surgery, as the healing period after OWHTO is approximately 6 months [32], and the patellar kinematic changes after 6 months were negligible in previous findings [9]. The stair height was set to 14 cm, which is one of the building safety standards used in North America [33]. The patients were asked to climb three stairs continuously under the surveillance of the DFIS (TAO image, Shanghai, China), whose accuracies reach approximately 0.1 mm and 0.3 deg [34,35]. The knee joint was imaged as the patients climbed the second stair using 30 pulsed snapshots per second (8 ms/pulse) by the DFIS. When the DFIS dynamic images were taken, a high-frame-rate force measurement table was arranged under the second step. The synchronized force signals were processed via a 5 Hz Butterworth low-pass filter (1000 Hz, Bertec, Columbus, OH, USA), and the heel-striking to toe-off moments of the subject’s foot were extracted as the motion cycle upstairs (Figure 1). The 3D surface model and 2D DFIS images were imported into customized software (MATLAB, 2023b, Boston, MA, USA), adjusting the models to match the bones’ outlines on each perspective image to reconstruct the spatial position of the bones during stair climbing. Furthermore, all patients underwent MRI scanning (3.0T, Philips, Amsterdam, The Netherlands) for OWHTO knees before and after surgery via a three-dimensional spoiled gradient echo sequence (3D-WATSc; repetition time (TR), 20 ms; echo time (TE), 7.5 ms; slice thickness, 0.5 mm; field of view, 100 mm; flip angle, 15°) to mark the attachment sites of the PT, MPTL, MPFL, and QMA. The CT images were input into Amira (Thermo Fisher Scientific, Rockford, IL, USA) to construct three-dimensional (3D) surface models of the femur, tibia, patella, and cartilage. The bone coordinate systems were built according to the definition recommended by the International Society of Biomechanics and previous studies [35,36].

**Figure 2:**
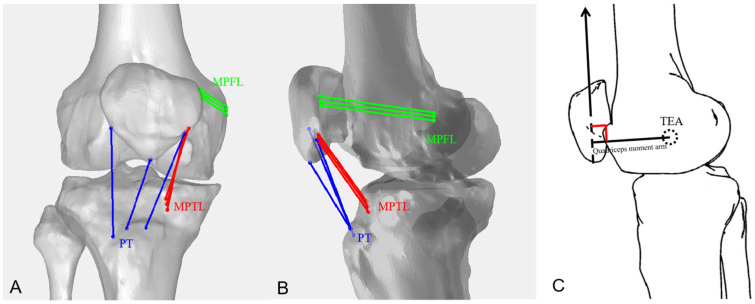
The line connecting the MRI bony insertions of the bundles was measured as the PT, MPTL, and MPFL bundle lengths during motion that were displayed on the frontal (**A**) and sagittal section planes (**B**). In addition, the length of the QMA was decided as the shortest (perpendicular) distance from the knee rotation center to the line connecting the footprints of the quadriceps muscles on the MRI (**C**).

Using MR images of each knee, the footprints of the PT, MPTL, MPFL, and QMA were digitized on the sagittal MR images. The insertion areas of the PT, MPTL, and MPFL were evenly divided into three equal portions to create three equal fiber bundles: the PTs were divided into the medial, central, and lateral bundles, whereas both the MPFL and the MPTL were divided into the proximal, central, and distal bundles. The length of each bundle was measured as the distance between the centroids of the bony insertions of the bundles from the series of matched knee models. Because the MPFL wraps around the medial femoral condyle, the line connecting the bundle centroids on the femur and patella was projected on the bony surfaces to create three curved lines (Figure 2A,B). The lengths of these projected curves were measured as the ligament bundle length. The direction of the quadriceps force was defined as the line connecting the footprints of the quadriceps muscles on the femur and patella, with the length of the QMA nearly equal to the shortest (perpendicular) distance from the knee rotation center to this connecting line at all flexion angles (Figure 2C).

The native knee is considered the ideal reference in current observation, as the difference in knee kinematics among individuals is significantly greater than that between the bilateral knees in humans [38], and no significant changes in the native side PFJ ligaments were found after OWHTO. We analyzed three groups in total to clarify the effects of OWHTO on the PT, MPTL, MPFL, and QMA: preoperative OWHTO, postoperative OWHTO, and native knee. After the Shapiro–Wilk test, we found that the experimental data were distributed non-normally, and all the measured parameters were tested for significant differences using the Wilcoxon rank–sum test. One-way ANOVA was used to analyze the significance of changes in the QMA before and after surgery. An unpaired two-tailed t test was used to analyze the significance of changes in the function scores. Pearson correlation analysis was used to determine the correlations between different types of data obtained in our study. A *p* value < 0.05 was considered to indicate a significant difference.

## 3. Results

**Figure 3:**
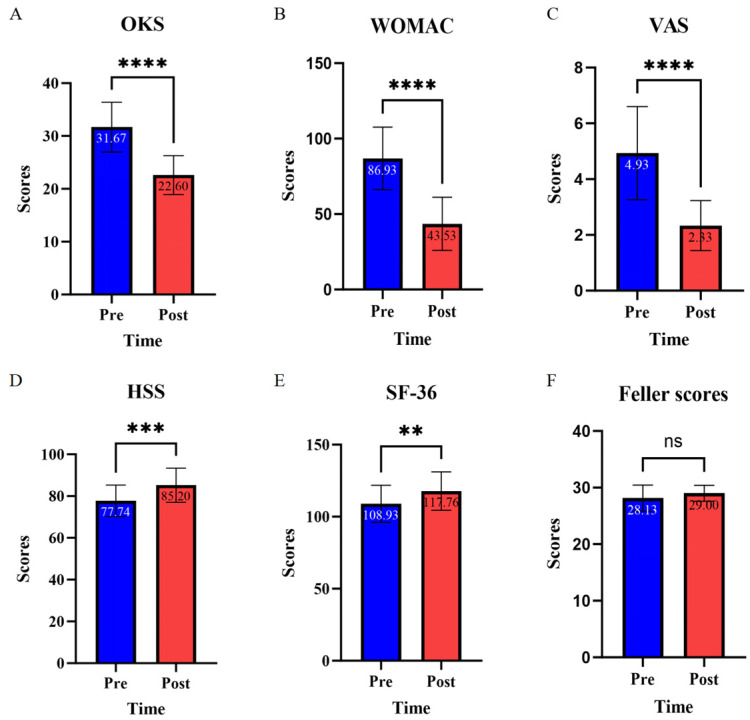
A function scoring system including OKS (**A**), WOMAC (**B**), VAS (**C**), HSS (**D**), SF-36 (**E**), and Feller scores (**F**) was used to compare the patient outcomes before and after OWHTO. The label ns means *p* value > 0.05, ** means *p* value < 0.01, *** means *p* value < 0.001, and **** means *p* value < 0.0001.

All surgeries were performed by a single senior surgeon, and osteotomy was performed to correct the weight-bearing line ratio (WBLR) passing through the position of the Fujisawa point (62.5% of the tibial plateau), which was mostly successful in achieving mild valgus knees and targeted clinical outcomes. Despite improvements in most function scores, the Feller scores showed no significant changes 6 months after surgery (*p* = 0.30) (Figure 3).

### 3.1. Ligaments and Tendons

#### Length Comparisons of Each PT, MPTL, and MPFL Bundle

**Figure 4:**
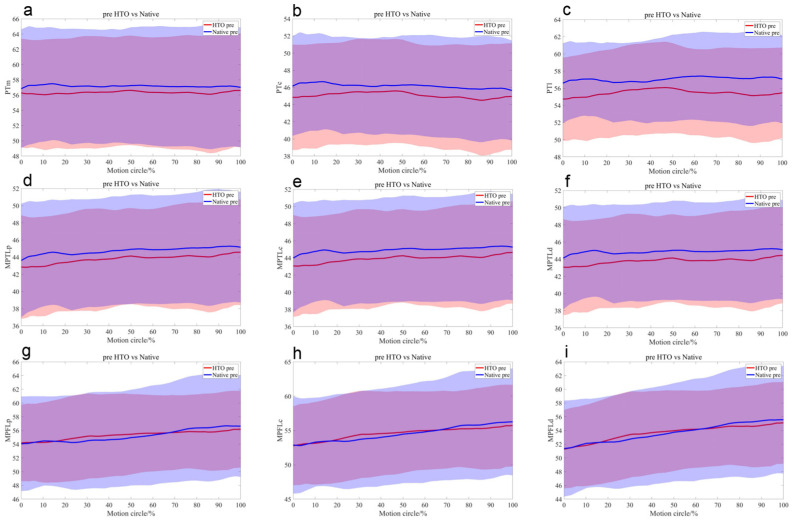
The mean length differences in the medial (**a**), central (**b**), and lateral (**c**) portions of the PT; the proximal (**d**), central (**e**), and distal (**f**) portions of the MPTL; and the proximal (**g**), central (**h**), and distal (**i**) portions of the MPFL during the stair-climbing motion with their standard deviations depicted using same-colour shading between the native (blue) and HTO (red) knees before surgery.

Preoperatively, the central (mean 1.6 mm, *p* = 0.01) and lateral (mean 1.7 mm, *p* < 0.01) PT bundles on the OWHTO side were significantly shorter than those on the native side. At the 20% motion cycle, during knee flexion, the lengths of the central and distal MPTL bundles on the OWHTO side were shorter than those on the native side (*p* < 0.05) (Table 1, Figure 4).

**Figure 5:**
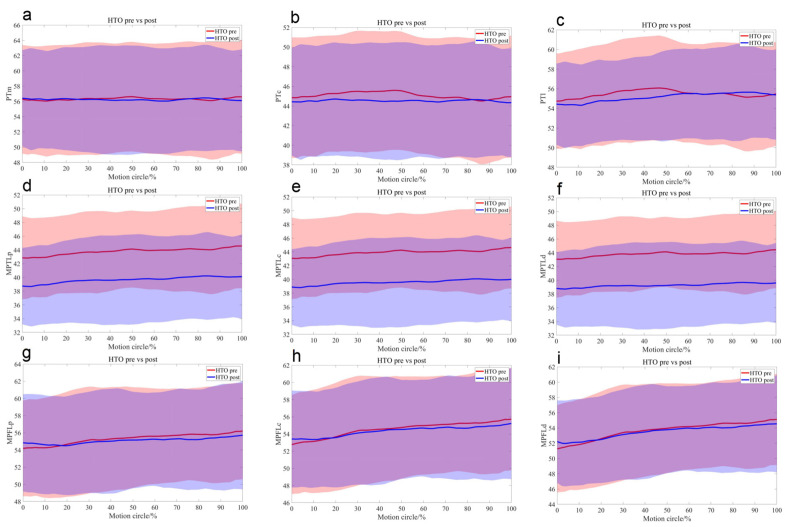
The mean length differences in the medial (**a**), central (**b**), and lateral (**c**) portions of the PT; the proximal (**d**), central (**e**), and distal (**f**) portions of the MPTL; and the proximal (**g**), central (**h**), and distal (**i**) portions of the MPFL during the stair-climbing motion with their standard deviations depicted using same-colour shading before (red) and after (blue) OWHTO.

Compared with the preoperative condition, all the MPTL bundles were significantly shorter (*p* < 0.001), with the PT and MPFL remaining statistically unchanged (Table 2, Figure 5).

**Figure 6:**
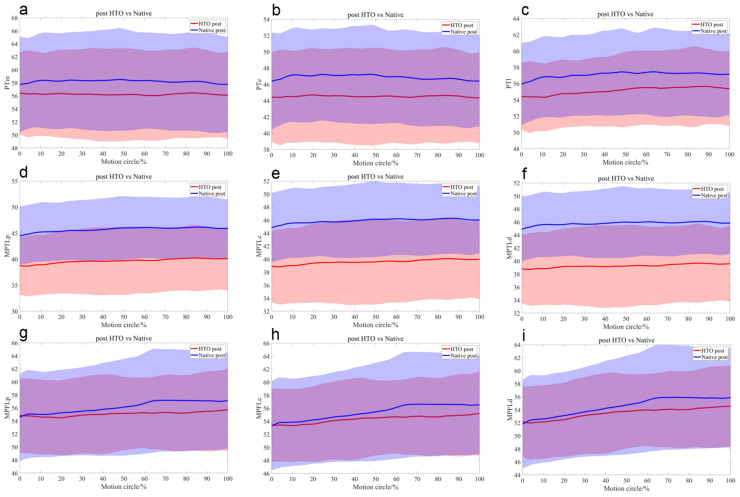
The mean length differences in the medial (**a**), central (**b**), and lateral (**c**) portions of the PT; the proximal (**d**), central (**e**), and distal (**f**) portions of the MPTL; and the proximal (**g**), central (**h**), and distal (**i**) portions of the MPFL during the stair-climbing motion with their standard deviations depicted using same-colour shading between native (blue) and HTO (red) knees after surgery.

Postoperatively, compared with those on the native side, the medial (mean 2.2 mm, *p* = 0.02), central (mean 2.4 mm, *p* = 0.01), and lateral (mean 2.2 mm, *p* = 0.03) PT bundles on the OWHTO side were significantly shorter, as were the MPTL bundles (*p* < 0.001). Moreover, there were no significant differences in the MPFL between the two sides (Table 3, Figure 6).

**Figure 7:**
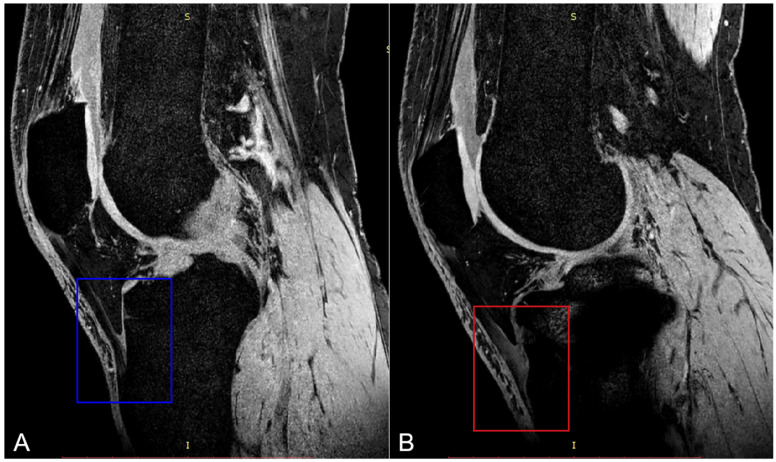
One of 15 patients’ knee MR images was randomly selected to display the typical changes happening in distal PT bundles at 6 months after OWHTO ((**A**) preoperative MRI; (**B**) postoperative MRI).

The MR images before and 6 months after OWHTO clearly revealed that the patellar tendon had adhered to the tibia at the osteotomy site (Figure 7).

### 3.2. Length Changes in the QMA

At early extension (first 20% motion cycle), the QMA significantly decreased by approximately 3.1% (mean 1.4 mm, *p* < 0.01) after OWHTO.

**Figure 8:**
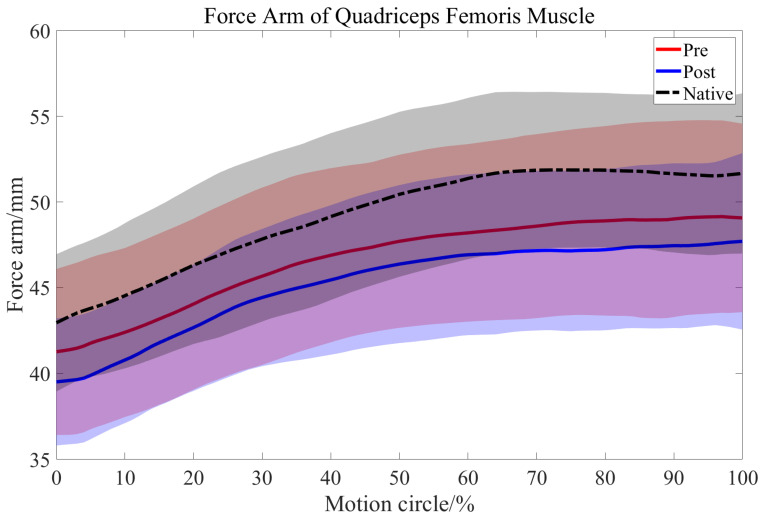
The mean length comparison among the QMAs on native side (black dashed line), preoperative OWHTO side (red line), and postoperative OWHTO side (blue line) during motion with their standard deviations depicted using same-colour shading.

Compared with the native side, the QMA on the OWHTO side was shorter by approximately 5.5% (mean 2.6 mm, *p* < 0.01) throughout the motion cycle, whereas OWHTO made it even worse, shorter than the native side by approximately 8.9% (mean 4.0 mm, *p* < 0.01) (Table 4, Figure 8).

### 3.3. The Correlation Between PT, MPTL, and the Open-Wedge Angle

Postoperatively, the differences in the PT and MPTL bundle lengths between the two sides were moderately related to the angle of the opening wedge (*p* < 0.05). The regression coefficients of the media, central, and lateral PT bundles to the open-wedge angle were −0.65, −0.62, and −0.56, respectively. For the proximal, central, and distal MPTL bundles to the open-wedge angle, the regression coefficients were −0.53, −0.52, and −0.52, respectively (*p* < 0.05). There was no relationship found between the other parameters after our systematic analysis.

## 4. Discussion

In the present study, we discovered that the preoperative lengths of the PT and MPTL bundles on the OWHTO side were shorter than those on the native side and were even worse after OWHTO. The PT and MPTL bundles decreased with increasing the open-wedge angle. All the MPTL bundles were shorter after OWHTO than before OWHTO, but there was no significant change in the MPFL bundle length. In addition, the QMA on the operative side was already shorter than that on the native side preoperatively, whereas OWHTO made it even worse. Furthermore, most function scores turned better 6 months after OWHTO, while the Feller scores changed insignificantly.

The functional changes in the MPTL and MPFL might cause the dynamic instability of the patella after OWHTO. The MPFL remained statistically unchanged after OWHTO, indicating that correction of the proximal tibia had few effects on the ligaments between the patella and femur. Furthermore, the reason for the shorter MPTL after surgery was easy to understand, as the distal patella was close to the proximal fragment of the tibia after OWHTO, the MPTL was becoming relaxed, which meant that the ligaments lost force to limit lateral patellar translation and valgus rotation at knee flexion [10,13]. Previously, MacIntyre et al. [5] revealed that the patella shifted more laterally in patients with AKP or PFJ malignment than in normal subjects, and Wilson, Nicole A. [39] reported the increased lateral patellar translation and valgus rotation in subjects with patellofemoral pain. Subsequently, the postoperative patients’ patellae might undergo abnormal movements once they perform daily knee flexions after OWHTO. As such abnormal movements tend to result in adverse clinical outcomes [40], the combined reconstruction of MPTL possibly needs to be investigated in further clinical studies.

The PT functional changes might be another cause of abnormal patellar kinematics after OWHTO. The PT was shorter on the operative side because of the more proximal position of the tibial tubercle related to the femur and patella in OA varus knees than in normal knees [41], which was also found in the preoperative condition of our observed knees. A previous in vivo study on PT changes during weight-bearing flexion in normal knees revealed that the length of all three PT portions sharply increased as the knee flexed from full extension to 30° and that the length of all three portions remained relatively constant between 30° and 110° [42]. As in the current study, the length of the PT showed no obvious fluctuations during the knee extension phase. In addition, Gokay et al. [15] reported that CWHTO might lead to adhesion of the distal PT on the proximal side of the osteotomy site, where severe vascularization, inflammation, and fibrotic changes are clearly observed, resulting in shortening of the PT and patellar descent. Another study reported that trauma generated systemic inflammation [43], together with cytokine proliferation, leading to increased fibroblast proliferation and increased vascularization. These factors, in turn, lead to increased and dysregulated collagen fiber deposition. Collagen fibers adhere to ligaments or tendons, causing contracture [44]. These findings matched the evidence of the distal PT adhesion to the osteotomy site on postoperative knee MRI, which, together with the distal shift of tibial tuberosity after OWHTO, explained the decrease in patellar height. In the present study, the PT of the OWHTO side was already shorter than that of the native side before surgery, but OWHTO made it even worse, which contradicted the previous assumption that the PT was elongated for the laterally and distally shifted tibial tubercle by OWHTO. In fact, the PT can hardly be stretched significantly longer in vivo because of its high elastic modulus [45].

It is feasible to minimize adverse PT changes by controlling the open-wedge angle or applying some physical therapies. After OWHTO, a moderate correction was found between the open-wedge angle and the differences in PT bundle lengths on the two sides. Specifically, the larger the open-wedge angle was, the shorter the postoperative PT was within a certain range. As closed trauma to the PT leads to partial cicatricial contracture of the PT and eventual patella infera [46], it is reasonable for doctors to limit surgical incisions and open-wedge angles during OWHTO. Furthermore, peritendinous adhesion is a postoperative complication after tendon injury, leading to poor knee function in the injured limbs [46,47], indicating that the tendon near the osteotomy site should be carefully preserved to avoid PT injury. Inspired by the treatments of adhesive capsulitis in other orthopedic designs [44], multiple treatment suggestions can be drawn as follows. (1) Antiadhesion barrier film [48] might be helpful for separating the osteotomy site and PT during surgery. (2) For patients who have already experienced postoperative AKP or dysfunction of the PFJ, ultrasound-guided therapies [49,50] or surgeries such as interposition of a pedunculated flap of the Hoffa fat pad [51] or Z-plasty lengthening of the PT [46] might work. (3) Finally, early training of the knee after HTO is essential for functional recovery, minimizing the shortening of the patellar tendon [52]. Therefore, early knee training as soon as a week after OWHTO is recommended for patients because the formation time of adhesion tissues is 5 to 7 days after injury, according to laboratory experiments [53]. Therefore, further clinical investigations are needed to compare the clinical outcomes of these suggestions and identify the best therapies.

The force on the moment arm of the quadriceps muscle was calculated during stair climbing before and after OWHTO because calculating the QMA could serve as a valid way to reflect the moment quadriceps forces and patellofemoral compressive forces [18] during knee extension. Previous observation reported that the temporary increase in PF contact forces was supposedly induced by postoperative short-term quadriceps atrophy [54]. In the current study, compared with that of the native side, the quadriceps moment arm of the OWHTO side was shorter (approximately 8.9%), which means that, according to the lever principle formula, the quadriceps muscle had to exert an extra 9.7% force to extend the knee compared with the native side. Compared with the preoperative situation, the QMA of the OWHTO side was shorter by approximately 3.1% in the first 20% of the motion cycle, meaning that the quadriceps muscle had to exert an extra 3.2% force to start extending the knee. The patellofemoral compressive force, as the resultant force of the quadriceps force and PT tension [55], may also increase by 3.2% after OWHTO. And it was reported that at larger flexion angles, the value of this reaction force is considerably greater [56]. These findings explain the degeneration of the PFJ in long-term follow-up and why surgical extremity needs more effort to accomplish motions demanding quadriceps forces after OWHTO. Therefore, to achieve balance between the two lower extremities, it is important to train the quadriceps muscles on the OWHTO side during postoperative rehabilitation, which aligns with the recommendation from previous rehabilitation research to perform quadriceps strengthening exercises within one year after OWHTO surgery [57]. Additionally, to deal with the increasing patellofemoral compressive forces, more attention is needed to monitor the changes in PFJ cartilage, and medicines such as glucosamine sulfate should be taken in the long term to prevent the progression of cartilage degeneration [58].

Several limitations of this study should be noted and explained. First, this study did not focus on the MPTL and MPML separately but rather focused on the MPTL as a sample. As the MPTL and MPML share a common patellar insertion [59] and their distal attachments are both located in the proximal fracture of the tibia after OWHTO, the MPTL and MPML show similar length changes in postoperative motion, which allowed us to simplify the observation. Furthermore, we considered only the stair-climbing movement, which is a type of middle-amplitude flexion movement. Other knee joint movements, such as running, walking, squatting, etc., have not been observed. According to previous studies, climbing stairs is the most demanding weight-bearing activity related to the PFJ [29] and is one of the most painful and challenging activities of daily living for subjects with anterior knee pain [30]. Therefore, we focused on the most effective daily activities related to the PFJ, excluding movements related to other joints.

## 5. Conclusions

The data revealed that the PT on the OWHTO side was shorter than that on the native side, and its length decreased with increasing the open-wedge angle, which was caused by postoperative adhesion. In addition, OWHTO led to the laxity of MPTL and the shorter QMA, which are adverse and deviate from the native leg, causing abnormal patella kinematics and imbalance between the lower extremities’ forces. In further studies, multiple clinical treatments should be purposely applied to optimize the surgery and rehabilitation.

## Figures and Tables

**Table 1:** The length differences in three ligament bundles between the two sides before OWHTO during the motion circle of stair climbing (mean ± SD).

Ligament	Bundles	0%	10%	20%	30%	40%	50%	60%	70%	80%	90%	100%
PT	M (mm)	−0.55 ± 3.43	−1.3 ± 3.24	−1.06 ± 3.32	−0.81 ± 3.35	−0.76 ± 3.48	−0.62 ± 3.57	−0.83 ± 3.13	−0.81 ± 3.15	−0.82 ± 3.02	−0.86 ± 2.96	−0.41 ± 2.94
	C (mm)	−1.31 ± 2.76	−1.63 ± 2.52 *	−1.11 ± 2.5	−0.76 ± 2.63	−0.69 ± 2.73	−0.73 ± 2.9	−1.18 ± 2.28	−1.18 ± 2.23	−1.11 ± 2.13	−1.25 ± 2.25	−0.7 ± 2.11
	L (mm)	−1.85 ± 2.04 *	−2.11 ± 2.63 *	−1.5 ± 3.05	−0.98 ± 3.26	−0.82 ± 3.31	−1.17 ± 3.35	−1.84 ± 2.61 *	−1.85 ± 2.32 *	−1.77 ± 2.25 *	−2.07 ± 2.55 *	−1.58 ± 2.7 *
MPTL	P (mm)	−0.77 ± 3.5	−1.47 ± 3.12	−1 ± 3.07	−0.77 ± 3.21	−0.9 ± 3.36	−0.79 ± 3.39	−0.93 ± 2.81	−0.92 ± 2.82	−0.96 ± 2.53	−0.98 ± 2.55	−0.59 ± 2.47
	C (mm)	−0.92 ± 3.35	−1.6 ± 2.96 *	−1.1 ± 2.88	−0.83 ± 3	−0.94 ± 3.13	−0.84 ± 3.19	−0.99 ± 2.63	−0.96 ± 2.65	−1 ± 2.4	−1.04 ± 2.45	−0.62 ± 2.37
	D (mm)	−1.06 ± 3.17	−1.7 ± 2.79 *	−1.19 ± 2.69	−0.9 ± 2.77	−0.99 ± 2.86	−0.9 ± 2.94	−1.07 ± 2.38	−1.02 ± 2.42	−1.05 ± 2.22	−1.11 ± 2.28	−0.67 ± 2.2
MPFL	P (mm)	0.12 ± 4.03	−0.22 ± 3.79	0.32 ± 3.6	0.66 ± 3.75	0.64 ± 3.47	0.49 ± 3.46	0.28 ± 4.09	−0.13 ± 4.14	−0.55 ± 4.13	−0.71 ± 4.34	−0.43 ± 3.58
	C (mm)	−0.11 ± 4.11	−0.16 ± 3.7	0.2 ± 3.56	0.7 ± 3.62	0.55 ± 3.32	0.29 ± 3.21	0.18 ± 3.93	−0.16 ± 4.13	−0.53 ± 4.1	−0.62 ± 4.29	−0.53 ± 3.7
	D (mm)	−0.1 ± 3.97	−0.29 ± 3.66	0.28 ± 3.37	0.66 ± 3.44	0.51 ± 3.19	0.22 ± 3.11	0.07 ± 3.83	−0.23 ± 3.99	−0.58 ± 4	−0.67 ± 4.33	−0.45 ± 3.67

* *p* value < 0.05.

**Table 2:** The length changes in three ligament bundles on the surgical side before and after OWHTO during the motion circle of stair climbing (mean ± SD).

Ligament	Bundles	0%	10%	20%	30%	40%	50%	60%	70%	80%	90%	100%
PT	M (mm)	0.14 ± 2.5	0.2 ± 3.01	0.18 ± 3.11	−0.11 ± 3.26	−0.23 ± 3.22	−0.45 ± 3.17	−0.26 ± 2.85	−0.12 ± 3.08	0.17 ± 3.19	0.04 ± 3.1	−0.47 ± 3.17
	C (mm)	−0.42 ± 2.49	−0.49 ± 2.86	−0.56 ± 2.94	−0.88 ± 3.11	−1 ± 3.21	−1.04 ± 3.08	−0.52 ± 2.66	−0.36 ± 2.81	−0.12 ± 2.96	−0.07 ± 2.99	−0.61 ± 3.03
	L (mm)	−0.31 ± 3.03	−0.63 ± 3.23	−0.55 ± 3.44	−0.9 ± 3.4	−0.97 ± 3.43	−0.72 ± 3.22	−0.05 ± 2.99	0.02 ± 3.03	0.18 ± 3.12	0.45 ± 3.24	−0.1 ± 3.32
MPTL	P (mm)	−4.11 ± 1.65 *	−3.99 ± 1.77 *	−4 ± 1.81 *	−4.1 ± 2.06 *	−4.2 ± 2.26 *	−4.44 ± 2.32 *	−4.17 ± 2.14 *	−4.08 ± 2.28 *	−3.94 ± 2.11 *	−4.11 ± 1.78 *	−4.44 ± 1.97 *
	C (mm)	−4.18 ± 1.62 *	−4.15 ± 1.79 *	−4.2 ± 1.8 *	−4.37 ± 1.96 *	−4.45 ± 2.21 *	−4.67 ± 2.23 *	−4.34 ± 2.05 *	−4.25 ± 2.25 *	−4.13 ± 2.12 *	−4.26 ± 1.79 *	−4.63 ± 2.03 *
	D (mm)	−4.26 ± 1.55 *	−4.32 ± 1.81 *	−4.4 ± 1.86 *	−4.62 ± 2 *	−4.7 ± 2.3 *	−4.9 ± 2.28 *	−4.54 ± 2.06 *	−4.44 ± 2.32 *	−4.33 ± 2.21 *	−4.43 ± 1.85 *	−4.83 ± 2.13 *
MPFL	P (mm)	0.65 ± 2.35	0.35 ± 2.57	−0.16 ± 2.21	−0.27 ± 2.05	−0.26 ± 2.27	−0.29 ± 2.18	−0.34 ± 2.19	−0.41 ± 2.26	−0.6 ± 2.34	−0.37 ± 2.6	−0.48 ± 2.4
	C (mm)	0.65 ± 2.48	0.22 ± 2.43	−0.09 ± 2.16	−0.27 ± 2.08	−0.21 ± 2.22	−0.22 ± 2.17	−0.29 ± 2.23	−0.33 ± 2.37	−0.57 ± 2.44	−0.47 ± 2.67	−0.5 ± 2.41
	D (mm)	0.94 ± 2.69	0.31 ± 2.45	−0.11 ± 2.12	−0.26 ± 2.08	−0.21 ± 2.26	−0.19 ± 2.18	−0.2 ± 2.32	−0.32 ± 2.38	−0.55 ± 2.46	−0.36 ± 2.65	−0.58 ± 2.41

* *p* value < 0.05.

**Table 3:** The length differences in three ligament bundles between the two sides after OWHTO during the motion circle of stair climbing (mean ± SD).

Ligament	Bundles	0%	10%	20%	30%	40%	50%	60%	70%	80%	90%	100%
PT	M (mm)	−1.33 ± 3.61	−2.13 ± 3.91 *	−1.91 ± 4.17	−2.09 ± 4.53	−2.22 ± 4.6	−2.32 ± 4.46 *	−2.27 ± 4.51 *	−1.99 ± 4.52	−1.77 ± 4.37	−1.74 ± 4.44	−1.64 ± 4.35
	C (mm)	−1.95 ± 3.41 *	−2.66 ± 3.55 *	−2.34 ± 3.7 *	−2.49 ± 4 *	−2.69 ± 3.98 *	−2.68 ± 3.81 *	−2.45 ± 3.91 *	−2.22 ± 4.01 *	−2.07 ± 3.88 *	−2.06 ± 3.84 *	−2.06 ± 3.76
	L (mm)	−1.51 ± 3.62	−2.48 ± 3.8 *	−2.01 ± 3.94 *	−2.12 ± 4.03 *	−2.28 ± 3.92 *	−2.13 ± 3.78 *	−1.88 ± 3.77	−1.83 ± 3.92	−1.72 ± 3.94	−1.58 ± 3.92	−1.77 ± 3.81
MPTL	P (mm)	−5.76 ± 2.25 *	−6.29 ± 2.64 *	−5.97 ± 2.98 *	−5.91 ± 3.51 *	−6.11 ± 3.78 *	−6.3 ± 3.68 *	−6.32 ± 3.94 *	−6 ± 3.85 *	−5.92 ± 3.39 *	−5.97 ± 3.15 *	−5.76 ± 3.16 *
	C (mm)	−5.95 ± 2.26 *	−6.54 ± 2.61 *	−6.23 ± 2.93 *	−6.2 ± 3.4 *	−6.4 ± 3.64 *	−6.54 ± 3.5 *	−6.54 ± 3.78 *	−6.23 ± 3.71 *	−6.18 ± 3.33 *	−6.21 ± 3.06 *	−6.01 ± 3.11 *
	D (mm)	−6.09 ± 2.3 *	−6.76 ± 2.65 *	−6.45 ± 2.97 *	−6.46 ± 3.41 *	−6.65 ± 3.62 *	−6.76 ± 3.45 *	−6.74 ± 3.73 *	−6.44 ± 3.68 *	−6.4 ± 3.35 *	−6.42 ± 3.07 *	−6.22 ± 3.14 *
MPFL	P (mm)	0.26 ± 3.35	−0.4 ± 3.16	−0.75 ± 2.83	−0.61 ± 2.95	−0.77 ± 3.32	−0.97 ± 3.95	−1.46 ± 3.85	−1.86 ± 3.82	−1.92 ± 3.62	−1.61 ± 3.47	−1.39 ± 3.66
	C (mm)	0.11 ± 3.45	−0.5 ± 3.1	−0.64 ± 2.73	−0.55 ± 2.78	−0.7 ± 3.14	−0.93 ± 3.86	−1.46 ± 3.73	−1.83 ± 3.76	−1.94 ± 3.58	−1.65 ± 3.46	−1.3 ± 3.63
	D (mm)	0.42 ± 3.63	−0.47 ± 3.3	−0.63 ± 2.83	−0.55 ± 2.88	−0.76 ± 3.27	−0.98 ± 3.95	−1.48 ± 3.82	−1.82 ± 3.85	−1.84 ± 3.68	−1.45 ± 3.51	−1.33 ± 3.82

* *p* value < 0.05.

**Table 4:** Two groups of the QMA comparisons during the motion circle of stair climbing (mean ± SD).

Comparison	0%	10%	20%	30%	40%	50%	60%	70%	80%	90%	100%
Pre vs. Post (mm)	−1.74 ± 2.47 *	−1.62 ± 2.68 *	−1.47 ± 2.95	−1.39 ± 3.77	−1.4 ± 3.59	−1.2 ± 3.85	−1.23 ± 3.87	−1.36 ± 3.64	−1.6 ± 3.33	−1.48 ± 3.18	−1.45 ± 3.47
Post vs. Native (mm)	−3.44 ± 2.73 *	−3.75 ± 3.23 *	−3.64 ± 3.86 *	−3.41 ± 4.24 *	−3.7 ± 3.94 *	−4.07 ± 3.96 *	−4.45 ± 3.83 *	−4.69 ± 3.51 *	−4.64 ± 3.61 *	−4.2 ± 3.88 *	−3.96 ± 4.41 *

* *p* value < 0.05.

## Data Availability

Data available on request due to restrictions eg privacy or ethical. The data presented in this study are available on request from the corresponding author. The data are not publicly available because the imaging data used for analysis is associated with the patients’ illness and privacy, and requires the patient’s consent before it can be used.
